# Is it possible to predict multidrug-resistant organism colonization and/or infection at ICU admission?

**DOI:** 10.1186/cc14180

**Published:** 2015-03-16

**Authors:** F Callejo-Torre, JM Eiros, S Ossa-Echeverri, P Olaechea, F Alvarez-Lerma, M Palomar, Envin-Helics Study Group

**Affiliations:** 1 Hospital Universitario de Burgos, Spain; 2 Hospital Clínico Universitario de Valladolid, Spain; 3 Hospital de Galdakao-Usansolo, Galdakao, Spain; 4 Hospital del Mar, Barcelona, Spain; 5 Hospital Arnau de Villanova, Lleida, Spain

## Introduction

We tried to develop a predictive model for patients colonized/infected by any multidrug-resistant organism (MDRO-C/I) at ICU admission based on risk factors easy to obtain (not depending on complex clinical records), being aware that foreseeing MDRO-C/I at ICU admission is key for appropriate empirical treatment and infection control.

## Methods

Data were collected prospectively from admission to discharge of 16,950 patients admitted consecutively (at least >24 hours) to 147 Spanish ICUs of the ENVIN (National Surveillance Study of Nosocomial Infections in ICUs) registry, from April to June 2010. To create the predictive model, 11,998 (2/3) patients were used for univariable and multivariable logistic regression model and 4,952 (1/3) for subsequent validation.

## Results

With a MDRO prevalence of 2.12% (359 MDROs at ICU admission were detected in 314 patients), 87.58% patients had only one MDRO, meanwhile 12.42% were MDRO-C/I by two or more simultaneously. Risk factors used in the development of the predictive model and independently associated with MDRO-C/I at ICU admission were (relative risk not shown due to space limitation): age 65 to 74, medical or surgical critical patient (especially urgent surgery), admitted from other ICU or long-term facility, immunosuppression and deep postsurgical skin or skin-soft tissue infections. Admitted from the community and female gender emerged as protective factors. Although the predictive model showed good discrimination (AUC-ROC = 0.775 (95% CI, 0.744 to 0.807)), sensitivity was only 67.4%. Validation with the remaining 4,952 patients (1/3) showed an AUC-ROC = 0.712 (95% CI, 0.665 to 0.759) and a *P *value on the Hosmer-Lemeshow goodness of fit test of 0.855. Even creating a new model, including variables obtained after ICU admission (severity by APACHE score, mechanical ventilation, central venous, arterial or urinary catheter, immunodeficiency, parenteral nutrition, ventricular derivation, extrarenal depuration, non-invasive ventilation, tracheotomy, enteral nutrition and nasogastric tube), prediction capability did not improve (AUC-ROC = 0.801 (95% CI, 0.774 to 0.828), sensitivity 71.4%).

## Conclusion

MDRO prediction at ICU admission could not be based merely on clinical-demographic risk factors. Taking into account local particularities and combining risk factors with a rapid laboratory test might be the most effective way forward.

